# A Visual Analytics Approach for Station-Based Air Quality Data

**DOI:** 10.3390/s17010030

**Published:** 2016-12-24

**Authors:** Yi Du, Cuixia Ma, Chao Wu, Xiaowei Xu, Yike Guo, Yuanchun Zhou, Jianhui Li

**Affiliations:** 1 Department of Big Data Technology and Application Development, Computer Network Information Center, Chinese Academy of Sciences, Beijing 100190, China; duyi@cnic.cn (Y.D.); xuxiaowei@cnic.cn (X.X.); zyc@cnic.cn (Y.Z.); 2 Intelligence Engineering Laboratory, Institute of Software, Chinese Academy of Sciences, Beijing 100190, China; cuixia@iscas.ac.cn; 3 Department of Computing, Imperial College London, London SW7 2AZ, UK; chao.wu@imperial.ac.uk (C.W.); y.guo@imperial.ac.uk (Y.G.)

**Keywords:** visual analytics, spatio-temporal visualization, time series visualization, multi-dimensional visualization, air pollution

## Abstract

With the deployment of multi-modality and large-scale sensor networks for monitoring air quality, we are now able to collect large and multi-dimensional spatio-temporal datasets. For these sensed data, we present a comprehensive visual analysis approach for air quality analysis. This approach integrates several visual methods, such as map-based views, calendar views, and trends views, to assist the analysis. Among those visual methods, map-based visual methods are used to display the locations of interest, and the calendar and the trends views are used to discover the linear and periodical patterns. The system also provides various interaction tools to combine the map-based visualization, trends view, calendar view and multi-dimensional view. In addition, we propose a self-adaptive calendar-based controller that can flexibly adapt the changes of data size and granularity in trends view. Such a visual analytics system would facilitate big-data analysis in real applications, especially for decision making support.

## 1. Introduction

Air pollution is becoming a pressing issue. A recent study [1] showed that approximately 3.2 million people died from air pollution-related causes in 2010 worldwide, 2.1 million of whom were from Asia. Additionally the number of paediatric patients in China with pneumonia has increased dramatically [2]. Air quality in China has become a hotly debated issue, and people want to be more informed about it. As a result, the Chinese government has been providing the public with air quality data from across the country.

Air quality data can be collected by different means, including monitoring stations and remote sensing satellites. Now, with pervasive sensing capability and deployment of large scale sensor infrastructure, we now have the capabilities to build “big” air quality datasets. Regardless of the method used, the collected data are usually spatio-temporal, and contain the location and time at which they were recorded. The data collected by monitoring stations are considered more accurate. Although each station can detect the air quality continuously, the published data often have different time granularities. For example, some stations may publish the data on an hourly basis, whereas others only release data a certain number of times per day. The stations are also distributed at different locations to detect air quality in specific areas. Further, the collected data are multi-dimensional and include values of NO_2_, SO_2_, PM_2.5_, PM_10_ and many other parameters.

The richness of the data collected by these stations offers opportunities for people to better understand air quality. In addition to knowing the current air quality, people can also analyze the trends, abnormalities and other interesting patterns about air quality. Researchers can conduct in-depth analysis to understand the causes and consequences of bad air quality.

However, the diversity of sensed data posts challenges for data analysis. In this paper, we propose a visual analysis system called AirVis to support the analysis of multi-dimensional spatio-temporal air quality data. With improved time series visualization methods and comprehensive interaction techniques, the system is aimed at helping people find more interesting patterns, which is very important to support decision making in real application, especially when the dataset has high volume and dimension. The main contributions of this paper are as follows:

1. AirVis, a visual analysis system, is proposed. This system can help the public and domain scientists find interesting patterns easily.
2. The new mechanism has comprehensive interactions and combines multi-dimensional visualization, spatio-temporal analysis, and multi-scale methods. This provides a general approach for big-data air pollution analysis.
3. A new adaptive development method is supported by multi-scale time series visualization and interaction.

The paper is organized as follows: Section 2 reviews related work from two different aspects, Section 3 describes the data source and the pre-processing of the dataset, and Section 4 is a system overview of AirVis. Then, we introduce the adaptive techniques of time series visualization in detail in Section 5. In Section 6, we give use cases to prove the usability of the system. Finally, we discuss some use cases and conclude the paper.

## 2. Related Work

### 2.1. Environment Related Visual Analytics

The environment is an essential facet in the development of society. It concerns many research areas [3,4], such as geography and ecology. Among this research, we can summarize some of the analysis tasks of environment-related domains: trends, abnormalities, cause, impact, and policies. A visual analytical system on these domains can help complete such tasks. Most of those systems are based on spatio-temporal datasets, and focus on different areas of the environment. EarthSystemsVisualizer (ESV) [5] and the systems proposed by Compieta [6] are two visualization systems developed to address large weather datasets. Both systems are task-based, and can help researchers complete analysis. HydroQual [7] is a system for the visual analysis of river water quality. It uses data collected by water quality stations. Compieta and HydroQual both incorporate data mining into their system, which can display mining results and spatio-temporal visualization. Vismate [8] is another visual analysis system for visualizing climate change. The system uses land surface observation data collected by meteorological observation stations. Similar to HydroQual, Vismate uses station-based data. In this system, three different visualization techniques are used to help analyze the long-term changes in climate. All of the visual analysis systems above can help in analyzing the environment. However, many of them cannot address multi-dimensional or multi-scale datasets well. Qu [9] proposed a visual analysis system for analyzing the air pollution problem in Hong Kong, which has a very similar domain to AirVis. AirVis uses a similar dataset as Qu, but the former is on a much larger scale and has different spatial and temporal granularities. It uses data collected by air quality monitoring stations. The dataset is updated hourly, and every data item contains multiple parameters.

### 2.2. Multi-Dimensional Spatio-Temporal Data Visualization

Because AirVis aims to address multi-dimensional and large-scale datasets, we investigate the related visual analytics techniques and interaction methods. The literature describes several spatio-temporal visualization techniques that help analyze spatio-temporal data [10,11,12,13,14]. Although they use different visual encodings, all these visualization techniques use a map as a basis. Qu [9] used parallel coordinates to analyze the multiple attributes of the air quality dataset so people can easily find the relationships between different attributes. Guo [15] proposed VIS-STAMP, a visualization system for space-time and multivariate patterns. Although both systems consider multi-dimensional analysis, the analyses are independent of spatial relationships. We cannot easily find the patterns behind the multi-dimensional data and spatial location.

There are also many temporal data visualization techniques used in spatio-temporal visual analytics. Aigner [16] reviewed some time series data visualization methods, and used that, some new techniques to visualize time series data [17,18]. Many of these methods do help in spatio-temporal visual analysis, however, when they are used in spatio-temporal visual analysis, they are usually used as a display control, and the interactions of the visual techniques and the interactions between spatial and temporal visualization are limited.

### 2.3. Multi-Scale Techniques in Spatial and Temporal Visualization

Multi-scale analysis is an important method for spatio-temporal visual analysis. It is similar to an interactive interface [19] that allows zooming, and the scalable analysis is reflected in both spatial and temporal facets. In spatial visualizations, we can analyze data by different continents [5], countries, regions [8] and stations [7]. Taking advantage of these systems, we let users select different spatial scales. In a time series visualization, as Aigner described [16], the time series data can be seen as linear time and cyclic time. The multi-scale analysis can reflect both facets. In addition to using different scales, time series data can be displayed at different levels of detail. The literature [20,21] discusses two time series visualization techniques, which use rectangular view for visualizing large time series data.

However, when time series visualization techniques are used in spatio-temporal analysis, we cannot pay attention on both points of view. Yuan [22] used cyclic rectangular view to visualize the pattern from different time of day, which is a similar techniques to [20,21]. However, the scale of time can be much more flexible, not just to analyze the cyclic pattern of different hours in days and weeks. [23,24], Landesberger [24] proposed a set of algorithms to help find the time steps, which is similar to our methods. However, the algorithm proposed here can be used in trend view, which has multiple time series charts. The methods fit Focus + Context well.

## 3. Data Source Materials and Methods

The method proposed in this paper is general for other air quality data from various sensor networks. As a case study, we adopted a dataset from collected at stations operated by the China National Environmental Monitoring Center (CNEMC) as a case study. CNEMC has 1437 stations across China. The number of stations of each province is shown in Table 1. CNEMC updates the air quality data every hour. The published data contains the value of SO_2_, NO_2_, CO, O_3_, PM_2.5_ and PM_10_. However, CNEMC does not provide past data. To analyze the air quality situation, we developed a web crawler, which can grab the hourly updated data and store them in a database. We began collecting data in December 2013. In this system, we use the air quality data from 1 January 2014, to 31 December 2014. In total, we have approximately12 million data items. Based on the names of the monitoring stations, we found all of the coordinates in the stations, which can be used on map-based visualization.

As described by CNEMC, the value of an item can occasionally be “null” when hardware or network problems occur. We also had several crashes of the crawler. To analyze the dataset efficiently, we pre-processed the raw data before the analysis. First, we cleaned the raw data. We found some null or obviously wrong (zero for example) monitoring values in the raw data and we removed such item from the raw dataset. Then, we mapped each data items with the space coordinates of its monitoring station. Finally, we generate a new dataset based on the processed dataset.

## 4. System Overview

We integrate three types of visualization techniques in this system. A map-based metaphor is used to visualize the distribution of stations and overall air quality situation. Trend view is used to visualize the trend of air quality items. Finally, calendar view is used to visualize the detail of different day and time circularly. When analyzing multi-dimensional data, we use multiple map-based visualizations and calendar views with one trend view. In each dimension, the color mappings of the map-based view and calendar view are the same.

### 4.1. Map-Based Metaphors

**Figure 1:**
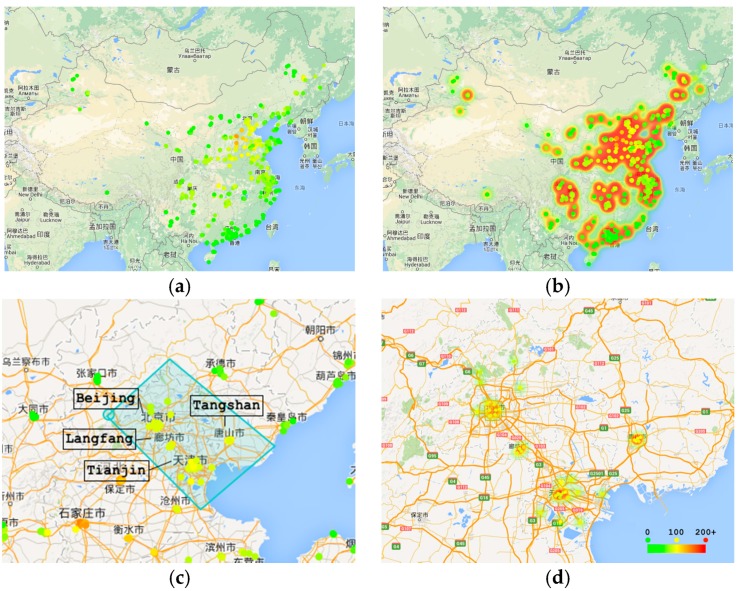
Map based views. Color from green to red means the air quality from good to bad. (**a**) Location of all the stations; (**b**) Overall situation of air quality in China; (**c**) Polygon selection tools used on the map. Beijing, Tianjin and several cities of Hebei Province are selected; (**d**) Detail of selected area.

We use Google Maps as our base map (Figure 1). Inside the map, there are two types of map-based metaphors for visualizing data. First, the points on the map indicate the location of stations. The colors of the points denote the average value during the year. Second, a heat map is designed to visualize the overall air quality of an area. Based on those two visualization techniques, we add interactions to support multi-scale-ey find.

### 4.2. Trends View

**Figure 2:**
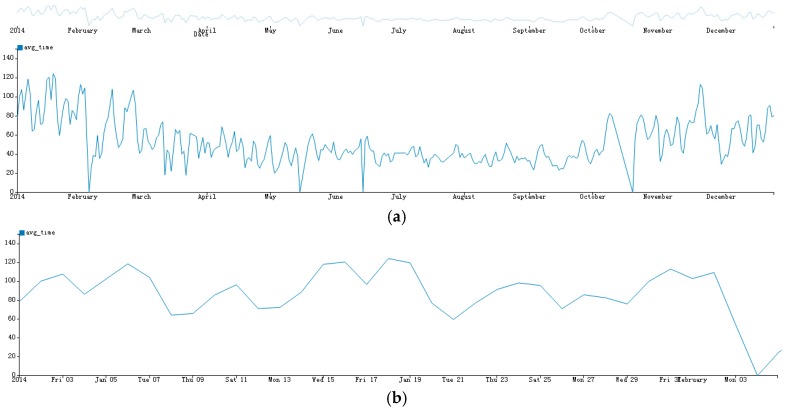
Trend view in AirVis. Plot (**a**) shows the initial trends view where the top and the bottom line chart have the same granularity; Plot (**b**) shows the focus view of the bottom line chart, which of it has the scale of hour, instead of day in the top line chart.

Line chart is a classic visualization method to display trends of different times. However, when the size of time varying data increases, effectively displaying all of the data in one line chart becomes a challenge. We developed a trend view based on the Focus + Context approach. The trend view is organized in two connected parts: On the top, there is an overall line chart that is used to visualize all data at a raw granularity; on the bottom, a detailed line chart is displayed. The overall line chart supports a brush to select a time period range, and the detailed line chart shows the detail of this period. In contrast to the traditional Focus + Context line charts, we select the granularity of both the top and the bottom charts automatically, which is useful for the dataset with different time granularities. The algorithm of the selection method is shown in Section 5. Figure 2 shows trend view at different time granularity.

### 4.3. Calendar View

A calendar view is often used to visualize periodical time series data. However, the periodical time series patterns must also reflect in hours, minutes or even seconds, which cannot be visualized in traditional calendar views. 

**Figure 3:**
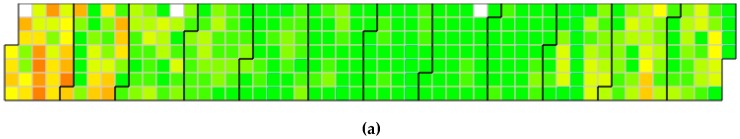

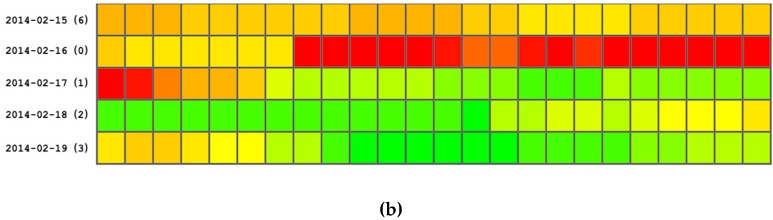
Calendar view in AirVis. Color from green to red means the air quality from good to bad. (**a**) Daily value of PM_2.5_ in several stations of China; (**b**) Hourly value of PM_2.5_ recorded by the same stations.

Therefore, we implemented a new calendar view that integrates the traditional calendar view and rectangular view to help finding multi-scale periodical timer series patterns. In this new view, a calendar is displayed to show the daily recorded air quality. With this calendar, we can find the average air condition of a given month, week and day. As shown in the top figure in Figure 3, there are 12 large blocks, each of which represents a month. When the time scale becomes small, the granularity of the calendar changes. A periodical rectangular view is used to discover the patterns of different days and hours. When the scale changes, the granularity of the time changes as well. As shown in the bottom figure in Figure 3, each line indicates a day of the selected time extent. The text on the left of each line shows the date, and the color shows whether the day is a weekday or weekend.

### 4.4. Multi-Dimensional View

**Figure 4:**
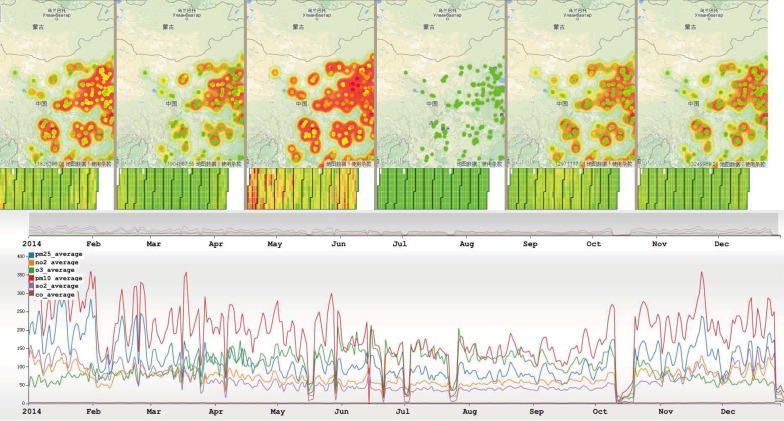
Multi-dimensional view in AirVis.

To support the visual analysis of multiple air quality parameters, we developed a multi-dimensional view. We did not choose ordinary multi-dimension visualization techniques such as scatter plots and parallel coordinates for several reasons. First, those techniques are independent of spatial relationships, so it is difficult find the patterns behind the multi-dimensional data and spatial locations. Second, there are at most six attributes except for the spatial and temporal attributes, and the amount of data is not sufficiently large to justify using high-dimensional visualization techniques. When analyzing using a multi-dimensional view, each parameter of the data uses a separate map-based chart and calendar view. Trend view uses multiple lines to display different attributes. As shown in Figure 4, the difference and relationship among the three parameters in both the spatial and temporal dimensions can be observed.

### 4.5. Interactions

**Figure 5:**
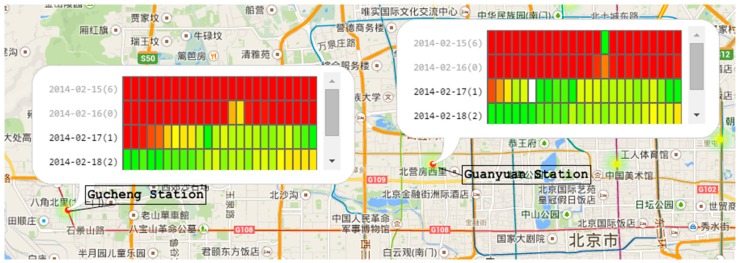
Tooltips on plots. Chinese words in the figure are POIs (point of interests) of a region in Beijing.

Interaction is very important when analyzing multi-dimensional data. AirVis incorporates some interaction tools to facilitate an analysis. First, a polygon selection tool is provided. As shown in Figure 1, this tool is used to select stations on the map, and users can find station patterns of interest. They can select stations by province, city, or even terrain. A brush tool on the context line chart is also available. When brushing on the context line chart, the adaptive algorithms find the best data of the time interval, and choose the best visualization techniques. Another tool is tooltips on plots. When analyzing the relationship and differences, users can click plots on the map. After clicking, a detailed calendar view appears to aid the analysis. Figure 5 shows two different stations in the calendar view of a specified time period.

## 5. Adaptive Multi-Scale Trend View

Time series data analysis requires two types of tasks: linear time analysis and periodical time analysis. In addition, there is a relationship between the granularities of time series data. For example, if the scale of the data is one year, users can analyze the linear pattern of seasons, months and days by different requirements. They can also analyze the cyclic patterns of a fixed time frame of a given day in a year, the cyclic patterns between weekdays and weekends, and so on. If the granularity of the dataset is much smaller, we can analyze patterns of different minutes, seconds, and even milliseconds, which is useful for analyzing different time series dataset.

**Figure 6:**
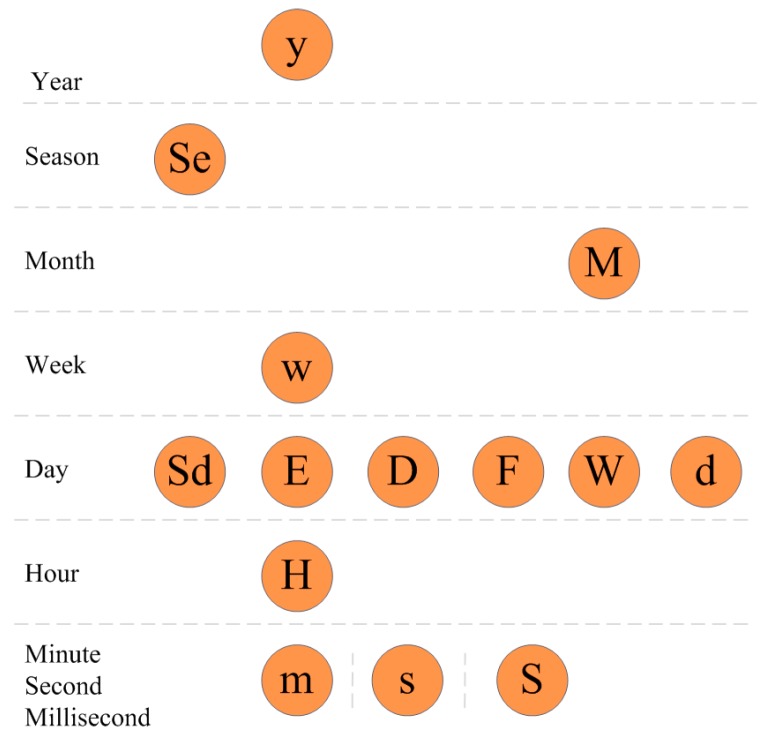
Level and granularity.

Motivated by the date and time structures in programming languages, we propose a novel design guideline of time series data. This guideline can address linear and cyclic time analysis. First, we define the granularity of time in Table 2. We also define nine time levels as year, season, month, week, day, hour, minute, second, and millisecond, as shown in Figure 6. For each granularity of time, there is a level where it belongs. For example, in level “Day”, we have Sd, E, D, F, W and d, which are all used to define “Day”, but have different granularities than the top level.

### 5.1. Linear Analysis Determination

We showed the granularity and level definition in Figure 6. For linear visual analysis, we should follow the nine levels as the granularity of the time series data. We define the scale between each level as an array *S*. The value of *S* is (1000, 60, 60, 24, 7, 4, 3, 4). Before designing the time series visualization, we define the following variables:

(1) Count the overall number of data items C.
(2) Define the minimum granularity of the time series data *g_min_*, which belongs to one of the nine levels.
(3) Determine the approximate display resolution of the screen R.

A trend view similar to that we propose for AirVis is very common in many visualization systems. The Focus + Context visualization approach is integrated with two line charts. Using Algorithm 1, we calculate the property granularity of the overview visualization. As described, the algorithm calculates the proper level of the result chart. The algorithm traverses from the minimum granularity of the time series data to the year, which is the maximum granularity of the data. During this period, the algorithm compares the overall number of data items in the visited level with the resolution of the display to find the property granularity of the overview view visualization. After running Algorithm 1, *g_max_* will return. Then, we can calculate the moment when the scale of the detail line chart changes, as shown below.

(1) $$\frac{\sqrt{R}}{\prod_{i=g_{min}}^{i<g_{max}}s\left[i\right]}$$

In the algorithm, *A* is a constant used to determine the threshold of the display.

**Algorithm 1**for *i←level(g_min_)* to *level(y)* do  if*((C←(C/S[i]))/*$\sqrt{R}$*)* < *A*  return*i as g_max_* end if end for return *level(y) as g_max_*

**Figure 7:**
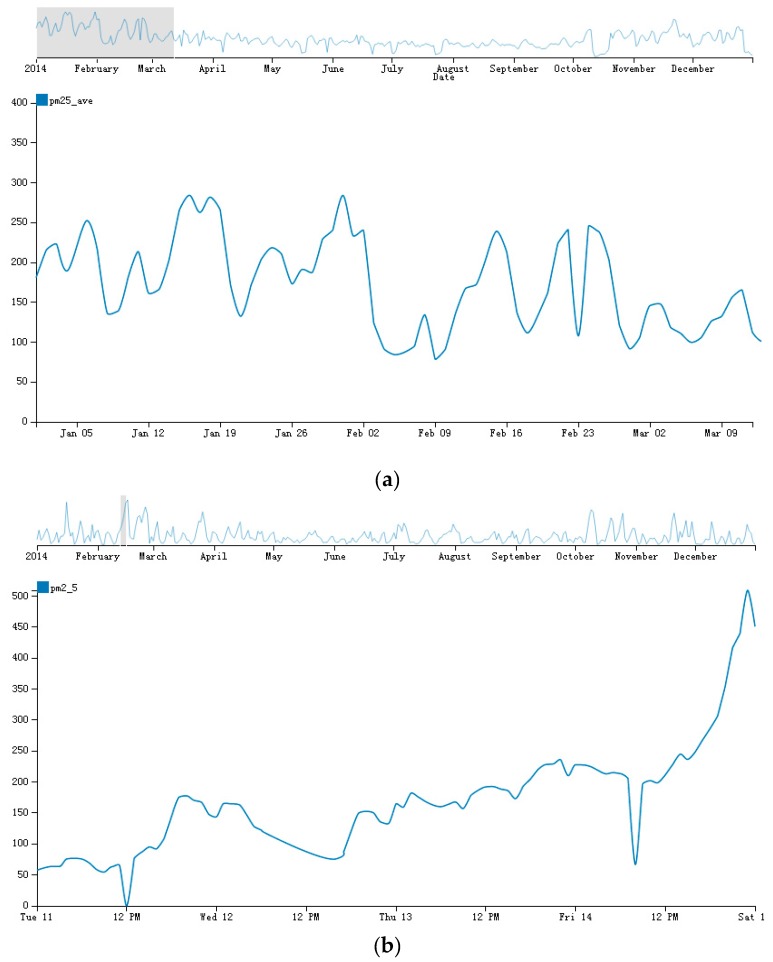
Trend view before and after running Algorithm 1. (**a**) Traditional Focus + Context view of trend view; (**b**) Focus + Context view using Algorithm 1.

We apply the algorithm to the air quality dataset. As described above, the minimum level of the dataset is “H”. Assume that we want to display the trends chart with two line charts on a 900 × 500 space, and apply Algorithm 1 to *A*. After running the algorithm, the result maximum level of the dataset is day. Thus, we can display the trend chart of each day. Additionally, the threshold of switching between the normal Focus + Context trend chart and the zoom-enabled one is 29, which means that if the extent of the brush is less than 29 days, the hour dataset will display on the focus view. As shown in Figure 7, when the brush extent is smaller than the threshold, the hourly data will display on the detail line chart.

The above algorithm and determination method are suitable when the time series visualization is integrated with two line charts with different granularities. We then extend the algorithm to a more common scenario, in which the number of line chart is *k*, and we use a recursive method to choose the best integration. First, we define the “best integration” as choosing the best integration from all the available levels. In this algorithm, we use the minimum variance of the scale of all selected levels as the “best integration”. The inputs of the algorithm are the minimum and maximum granularities calculated by Algorithm 1, and the number of line charts. In one recursion, the algorithm estimates whether the number of charts is equal to *k*. If so, the variance is calculated to determine whether it is the best integration. If not, a loop from the level of small granularity to level of large granularity is executed, in which the recursion method is invoked and the scale of each selection is calculated. The algorithm is shown as Algorithm 2.

**Algorithm 2**define *min* as a large number end define define *cal(g_small_, g_big_, n)*  if *n* equals *k*  cal the variance of r  if *r* < *min* *min ← r* end if  end if  if *n* < *k*  for i * ←level(g_small_)* to *level(g_big_)*  r[n] *←*$\prod_{i=g_{small}}^{i<g_{big}}S\left[i\right]$  nLevel[n] *←i*  *cal(i, g_big_, n)* end if end define call cal(level(*g_min_*, *g_max_*, 0)) return nLevel

We give several examples of the algorithm’s usage. Table 3 gives several examples of the nLevel of different groups of number of charts and granularity boundary.

### 5.2. Cyclic Analysis Determination

**Figure 8:**
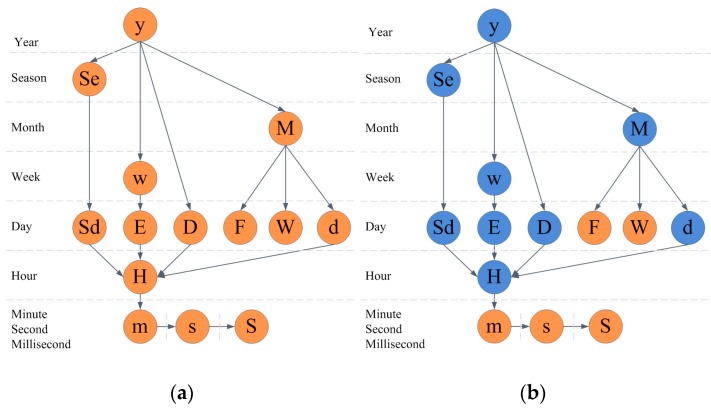
Cyclic analysis graph. (**a**) Level and granularity with analysis relationships; (**b**) All the cyclic patterns in the Level and granularity graph.

Based on the linear method, we give the cyclic analysis determination. We add arrows to Figure 6 when the relationship of each level is known. For example, we add an arrow from “y” to “D”, which means that we can analyze the cyclic pattern of the same day. Then, a directed acyclic graph is constructed, as shown in Figure 8a. An arrow indicates that the cycle is allowed, such as when the minimum granularity of the time series data is hour, and the scale of the data is larger than one year. A sample of the cyclic patterns we can analyze is as follows:

“First, we can analyze the cyclic pattern of weeks in several years. Then, we can also analyze the cyclic pattern of days in several weeks. Finally, we can analyze the cyclic pattern of hours in several days”.

Similar patterns of the first step are months in several years, seasons in several years, days in several years, while similar patterns of the second step are days in several months, days in several seasons, hours in several days. Figure 8b is all the cyclic patterns we can analyze. The blue circles of the graph construct all the cyclic patterns.

**Figure 9:**
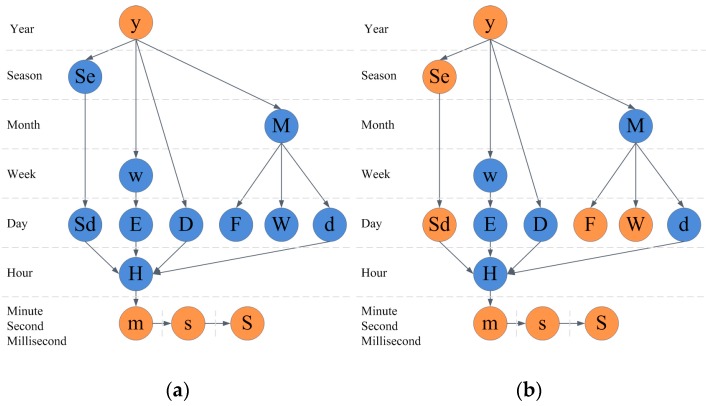
Cyclic analysis graph in AirVis. (**a**) All supported cyclic patterns of the air quality dataset; (**b**) All supported cyclic patterns in AirVis.

If we take the air quality dataset, for example, the scale of the data is one year, so the cyclic patterns that we can analyze are shown in Figure 9a. The patterns that we support in AirVis are shown in Figure 9b.

## 6. Case Study

### 6.1. PM_2.5_ Analysis

**Figure 10:**
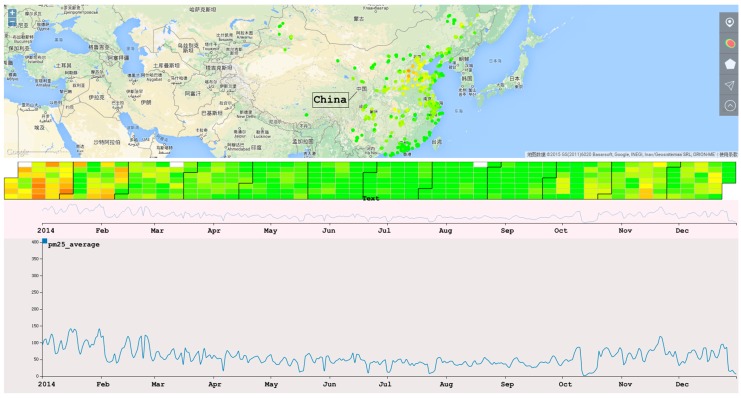
The overall view of AirVis. Chinese words in the figure are country and city names in this regions, which will not affect the understanding of the figure.

**Figure 11:**
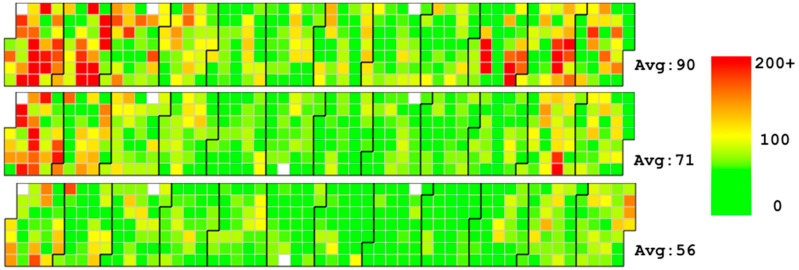
Daily trends of different area in China. Top: Daily trends of Beijing, Tianjin and Hebei province. Middle: Daily trends of Shandong Province. Bottom: Daily trends of Yangtze River Delta.

First, we analyze one of the most important indices of air quality, PM_2.5_. As shown in Figure 10, the interface of the system includes a map-based view, a calendar view and a trend view. Using this system, we can find some general information:

(1) The overall distribution of the stations. We can see that Eastern China has more stations than Western China and that most of the stations are placed in large and medium-sized metropoli, such as Beijing.
(2) The initial visualization shows the overall situation of PM_2.5_ in 2014. From this, we find that northern China has a higher average value of PM_2.5_ than other areas of China.
(3) From the calendar view, we find that spring and winter have notably higher concentration of PM_2.5_ than summer and autumn. From the line chart, we find some interesting patterns in addition to the seasonal differences. We find that although the overall trends exist, the values of neighboring days change significantly.
(4) AirVis supports the flexible selection of areas and stations. By analyzing the daily trend of different areas in China, shown in Figure 11, we find that the daily value of PM_2.5_ of Beijing is higher than in the Shandong Province and the Yangtze River Delta. The situation in the Yangtze River Delta during spring time is slightly better than that in the Shandong Province.

**Figure 12:**
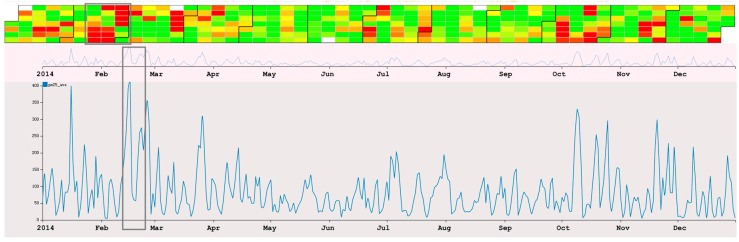
The daily trends of PM_2.5_ in Beijing. The selected area shows one of the interesting patterns we found.

**Figure 13:**
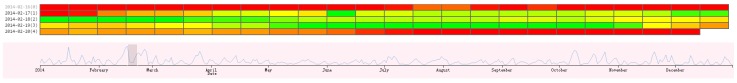
The hourly trends of PM_2.5_ in Beijing from 16 February to 20 February. This shows the detail change of the interesting pattern.

Then, we focus observation to Beijing. There are 12 stations in Beijing. By selecting all 12 stations on the map, we can analyze the air quality in Beijing. We also find some interesting patterns:

(1) There are similar trends between Beijing and the rest of China. Stations record higher concentration of PM_2.5_ during spring and winter than during summer and autumn. However, Beijing has much higher averages.
(2) There is a very interesting patterns of the value of PM_2.5_ from 13 February to 28 February. As we can see in Figure 12, the value of PM_2.5_ changes from a high value to a low value. After three days of low value (17th–19th), the value rises again (20th–26th). After the 26th, the value reduces to a low value again. To our knowledge, the reason for the rise and fall of PM_2.5_ value because of wind in the city. However, the value reduces following the 17th, and there is no wind during the period. The detail of the hourly value of those days can be seen in Figure 13, and we can see that the change of the value is a gradual process.

### 6.2. Multi-Dimensional Analysis

**Figure 14:**
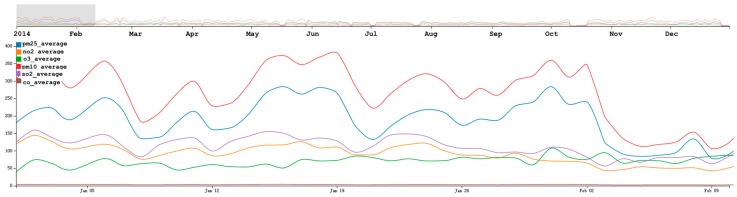
Daily trends of six attributes in China. As shown in the figure, the blue line represents the value of PM_2.5_, the orange line represents the value of NO_2_, the green line represents the value of O_3_, the red line represents the value of PM_10_, the purple line represents the value for SO_2_, and the brown line represents for the value for CO.

Similar to the PM_2.5_ analysis process, we first provide a general overview of the parameters in the air quality dataset. As shown in Figure 14, the overall trend of the selected six attributes are similar, especially for the value of PM_2.5_ and PM_10_. When analyzing the six attributes in Beijing, we found the average daily trends of PM_2.5_ and PM_10_ are similar. As described in Section 5.1, we found that the value of PM_2.5_ decreased starting on 17 February, which was very strange. As shown in the multi-dimensional view, we found that the value of PM_10_ also had a similar decreasing trend. By comparing the values of PM_2.5_ and PM_10_ using AirVis, we found that there are two significant high values of PM_10_ on 24 February to 25 February, and on 17 March to 18 March. However, the value of PM_2.5_ from 24 February to 25 February was high, whereas that from 17 March to 18 March was low.

### 6.3. Domain Experts Feedback

Our system was assessed by two domain experts of Chinese Academy of Sciences. Their expertise included in areas of regional air pollution, indoor and urban air pollutants. During the consultation, we first discussed the domain requirements. Then we provided our system to them and allowed them to explore the air quality data using our system. We collected the feedback on both the usability of the system and the explanation of the result.

For the system itself, both the two researchers gave positive feedback about the exploration process of our system. In their traditional way of doing research on air quality, they have to use different tools to help them generate different visualizations. One of a time consuming tasks is to convert data from one tool to another. They found our system to be particularly helpful as it integrates multiple visualizations that can generate visualizations at different time ranges and spatial regions in real time. One expert mentioned that the linked view is very helpful for exploring the data, especially for the link between the calendar view and the trend view. The other expert was interested in the zoomable map with heat map, which provided a flexible interaction. One researcher was particularly eager to explore his own data using our system. However, our system did not support the capability to interactively ingest new data sources. We leave this as future work. The researchers also gave some suggestions on optimizing the system. They said there were many clutters when they analyzed in multi-dimensional view. They commented that the overall patterns and anomalies found using the system were helpful clues for future analysis. They also suggested integrating other data sources into our system (e.g., traffic, atmospheric variables) to further explore potential patterns of air pollution.

## 7. Discussion

After finding the patterns using AirVis, we attempted to analyze their causes. Some of the changes in air quality are obvious, such as the seasonal changes of PM_2.5_ concentration. Some findings further prove the theories that we previously established, such as the functions of the wind on the air quality.

The PM_2.5_ values in Northern China are very high. The reasons are complex, but the industrial structure and terrain are important possible explanations. When digging into one specific area, we found that the value of PM_2.5_ has increased gradually since 20 February. This is mostly due to the working of the city. When there is not sufficient wind to blow PM_2.5_ out of the city, and the city itself generates PM_2.5_ gradually, this causes an increase of PM_2.5_.

We also observed that the recorded values of PM_2.5_ from 17 to 18 March were low, whereas the multi-dimensional data analysis shows that the value of PM_10_ during those two days was very high. By looking at the weather during those days, we found that there was a strong north wind during those days. The wind blew PM_2.5_ away and brought in PM_10_ from the northern part of China.

Similarly, we found that the value of PM_2.5_ started to decrease on 17 February. However, when analyzing the multi-dimensional view, we found that the value of PM_10_ was also low, which is different from the days in the previous case. The weather of those days was very calm. The experts were also confused by this pattern. They suggested several possible explanations: (1) the source of PM_2.5_ decreased in the city (e.g., some events caused the number of the moving cars to decrease); (2) the data proposed by CNEMC were wrong; or (3) some other undiscovered factor affecting PM_2.5_ caused the situation.

By analyzing the data multi-dimensionally, we found that the values of PM_2.5_, PM_10_ and other air quality attributes have some relationships. Wind occasionally caused an increase of PM_10_ and a decrease of PM_2.5_, but at other times, the value of PM_2.5_, PM_10_ and other air quality attributes were positively correlated.

## 8. Conclusions and Future Work

In brief, this paper present AirVis, a visual analytical system for air quality analysis. In the system, three different views are integrated. A map-based view is used to analyze the spatial distribution of stations and the situation of different areas. A calendar view gives users insight into the cyclic situation of air quality. This view is designed as an Overview + Detail calendar view. When interesting patterns are discovered in the map-based or trend views, we can dig into the data to find the cyclic trends for every hour per day. A trend view can display the quality trend by two line charts. Similar to the calendar view, trend view is also connected by two relevant parts. These two parts are designed with a Focus + Context approach.

Motivated by programming language, we improve the trend view by providing nine levels to describe time. There are several granularities in each and based on the methods and the graphs generated by the levels, we give design guidelines to help design visualizations to indicate linear and cyclic trends of time-varying datasets. In addition, we also give algorithms to determine the granularities and the moment when scale should change. However, although we proposed algorithms and provided some use cases and the results, we did not note when the description of level changes in scale and granularity. In our discussion, we believe that only the definition of the level and the scale array change can solve the problem. The algorithms proposed need not change. In future works, we will find additional datasets to prove the hypothesis.

Using AirVis, we gave two use cases: an analysis of the PM_2.5_ situation in China and a study of six attributes of air quality data. During the analysis, we found some interesting patterns that were not easy to find otherwise. These findings can help scientists analyze changes in air quality. When using AirVis to analyze multi-dimensional air quality datasets, we use separate map-based views and calendar views, instead of using parallel coordinate or other multi-dimension data visualization methods. The reason is that the maximum number of dimensions of the air quality dataset is six. However, evaluation on those two methods is also necessary in future work. In Section 7, we discussed some causes for the patterns we found using AirVis. Among those causes, we found that air quality is correlated to natural factors such as wind patterns and temperature. Additionally, in some research on the cause and impact of air pollution, many other types of datasets such as land usage, economic development and car ownership data, are used. However, AirVis does not support visual analysis of these causes. In future work, we will extend AirVis’ support for visual analysis of correlation between air quality data and other datasets.

As mentioned by the domain experts, the exploration process of the system could do great help to find patterns. The analysis results are also very interesting, and could be a good supplement to current research on air quality. However, the cause and influence of air pollution is related to many factors [25,26,27], such as wind, temperature, land use, emission of pollutants. In order to increase the flexibility, we will support the capability to interactively ingest new data sources. We will also try to include additional data sources to further explore potential causal relationships of air quality (e.g., traffic) in the future work.

## Figures and Tables

**Table 1:** Provinces and number of stations.

Province	Number of Stations	Province	Number of Stations	Province	Number of Stations
Beijing	12	Tianjin	14	Chongqing	17
Shanghai	10	Inner Mongolia	44	Liaoning	78
Jilin	33	Heilongjiang	57	Shanxi	58
Jiangsu	72	Zhejiang	47	Anhui	68
Fujian	37	Jiangxi	60	Shandong	74
Henan	75	Hubei	51	Hunan	78
Guangdong	102	Guangxi	50	Hainan	7
Hebei	53	Sichuan	94	Guizhou	33
Yunnan	40	Tibet	18	Shanxi	50
Gansu	34	Qinghai	11	Ningxia	19
Sinkiang	41				

**Table 2:** 9 Levels and Their Descriptions.

Level	Granularity	Description
Year	y	Year
Season	Se	Season in year
Month	M	Month in year
Week	W	Week in month
	w	Week in year
Day	D	Day in year
	Sd	Day in season
	d	Day in month
	F	Day of week in month
	E	Day in week
Hour	H	Hour in day
Minute	m	Minute in hour
Second	s	Second in minute
Millisecond	S	Millisecond in second

**Table 3:** 9 Levels and Their Descriptions.

*g_min_*	*g_max_*	Number of Charts	nLevel
m	M	3	[m, H, M]
s	Y	3	[s, h, Y]
s	Y	4	[s, m, d, Y]
s	Y	5	[s, m, h, w, Y]
